# The predictive value of immune inflammation indexes for the risk of fracture in patients with osteoporosis: a systematic review and meta-analysis

**DOI:** 10.3389/fendo.2025.1650895

**Published:** 2025-11-03

**Authors:** Yawei Xu, Xiaoming He, Xu Zhang, Shilong Meng, Chengjie Wang, Yifeng Yuan, Xiaolin Shi, Kang Liu

**Affiliations:** ^1^ The Second Clinical School, Zhejiang Chinese Medical University, Hangzhou, Zhejiang, China; ^2^ The Second Clinical School, The Second Affiliated Hospital of Zhejiang Chinese Medical University, Hangzhou, Zhejiang, China

**Keywords:** osteoporosis, osteoporotic fracture, immune inflammation index, meta-analysis, predictive value

## Abstract

**Background:**

This study aims to elucidate the value of immune inflammation indexes like neutrophil-to-lymphocyte ratio (NLR), systemic immune-inflammation index (SII), lymphocyte-to-monocyte ratio (LMR), platelet-to-lymphocyte ratio (PLR), as well as systemic inflammation response index (SIRI) in fracture risk prediction among the osteoporosis population and the extent of their association.

**Methods:**

PubMed, Embase, Web of Science, the Cochrane Library, CNKI, as well as Wanfang were thoroughly retrieved until March 22, 2025. The primary outcome measure was the link of the immune inflammation indexes to fracture risk in osteoporotic people. The consistency of findings and possible origins of heterogeneity were examined via sensitivity and subgroup analyses. Data analysis was enabled by STATA 18.0 and Review Manager 5.4.

**Results:**

This meta-analysis encompassed eight trials on 3,769 participants. The summary findings indicated that in studies considering categorical variables, NLR (odds ratio (OR) =1.73, 95% confidence interval (CI): 1.40-2.14; p<0.00001), LMR (OR = 0.85, 95% CI: 0.77-0.93; p=0.0007), as well as SII (OR = 1.01, 95% CI: 1.00-1.01; p=0.008) had a significant link to fracture risk in the osteoporosis group, while no such correlation was seen for PLR (p=0.10) and SIRI (p=0.32). In continuous variable analyses, all indexes exhibited a significant connection with the likelihood of fracture. The subgroup analysis indicated that the classification of osteoporosis may affect the prognostic performance of immune inflammation indexes.

**Conclusion:**

The immune inflammation index NLR, LMR, and SII possess substantial predictive value for fracture risk in osteoporotic individuals. Moreover, the association between SIRI and fracture risk should be interpreted with caution, as it is derived from only two studies. Nevertheless, several limitations must be acknowledged: most included studies adopted retrospective designs, the study populations were predominantly Asian, and issues such as potential publication bias and result instability cannot be excluded. Therefore, further high-quality investigations are warranted to substantiate our findings.

**Systematic review registration:**

PROSPERO, identifier CRD420251053366.

## 1 Introduction

Osteoporosis is the most prevalent systemic skeletal illness featuring reduced bone mineral density, bone micro-structure destruction as well as elevated likelihood of fracture (1). Investigations from pertinent institutes indicate that nearly 33% of women and 20% of men over 50 face the risk of developing osteoporosis globally (2). The rapid aging of populations across the world will likely lead to a substantial growth in osteoporosis prevalence, consequently raising fracture occurrence rates. Fragility fractures are a frequent complication and significant clinical outcome of osteoporosis usually arising from low-energy external stresses (3, 4). It can profoundly lower the quality of life and health by inducing considerable pain, disability, loss of independence, and potentially mortality in affected individuals (2). Therefore, the efficient and prompt prevention of osteoporosis and the associated fracture risk has emerged as an essential priority in contemporary public health (5). In contemporary clinical practice, the assessment of osteoporotic fracture risk primarily relies on bone mineral density measurements and predictive models such as FRAX (6). Traditional predictive approaches incorporate selected clinical risk factors but overlook systemic inflammation, a pivotal pathophysiological mechanism in osteoporosis development (7). In contrast, immune inflammation indexes derived from routine complete blood counts serve as dynamic indicators of the body’s inflammatory status. These biomarkers are cost-effective, readily accessible, and capable of providing real-time reflections of immune-inflammatory activity. By incorporating inflammation as an independent risk determinant, such indices can substantially augment existing assessment models, offering a novel biological perspective for identifying high-risk individuals who may otherwise be underestimated by the FRAX model.

Inflammation is strongly linked to the onset of varied chronic diseases arising from proinflammatory cytokine secretion and persistent immune system activation (8, 9). Related studies suggested that changed host immunological status is an important danger factor contributing to abnormalities in bone metabolism, with its indirect effects potentially causing persistent bone damage (10, 11). This possibly results from the immunological microenvironment, where various immune cells interact with osteoblasts to yield outcomes (12). The immune inflammation index is a composite metric derived from peripheral blood cell subset counts for thorough inflammatory response and immune status evaluation. It reflects the dynamic interplay between circulating immune cells and inflammatory markers. Commonly utilized indexes encompass the neutrophil-to-lymphocyte ratio (NLR), systemic immune-inflammation index (SII), lymphocyte-to-monocyte ratio (LMR), platelet-to-lymphocyte ratio (PLR), as well as systemic inflammation response index (SIRI), among others. In the domain of osteoporosis, an increasing number of studies on immune inflammatory indexes are being carried out, with the expectation of offering novel approaches for predicting fracture risk in affected populations. Fu et al. identified NLR, MLR, as well as SIRI as independent spinal compression fracture risk factors, with MLR demonstrating a comparatively high diagnostic capacity (13). Fang et al. proved SII as an effective risk predictor for postmenopausal osteoporosis evaluation and a reliable discriminator of osteoporotic fracture risk (14).

To date, many clinical studies have validated the prediction value of immune inflammatory indexes concerning fracture risk. However, the existing evidence remains limited, with insufficient sample sizes and a lack of comprehensive analyses. Notably, no systematic review has yet been published to elucidate the clinical significance of this topic. Therefore, the present study aims to systematically evaluate and meta-analyze the available clinical data to determine whether immune inflammatory indexes can serve as effective fracture risk predictors in the osteoporosis population. Moreover, this study endeavors to provide an updated theoretical foundation to support more accurate fracture prediction model development for clinical application.

## 2 Materials and methods

### 2.1 Literature search

Our study followed the Preferred Reporting Items for Systematic Reviews and Meta-Analyses (PRISMA2020) statement (15). The protocol was registered on the International Prospective Systematic Evaluation Registry (PROSPERO: CRD420251053366). PubMed, Web of Science, Embase, Cochrane Library, CNKI, as well as Wanfang were comprehensively retrieved until March 22, 2025. Subject and free-text terms were used and included: “Lymphocytes”, “Lymphocyte”, “Lymphoid Cells”, “Lymphoid Cell”, “Ratio”, “Fractures, Bone”, “Fracture”, “Bone Fracture”, “Bone Fractures”, “Broken Bones”, “Broken Bone”, “Spiral Fractures”, “Spiral Fracture”, “Torsion Fractures”, “Torsion Fracture”, “Osteoporosis”, “Osteoporoses”, “Age-related Osteoporosis”, “Age-related Osteoporoses”, “Age-related Bone Loss”, “Age-related Bone Losses”, “Senile Osteoporoses”, “Senile Osteoporosis”, “Post-traumatic Osteoporoses”, and “Post-traumatic Osteoporosis”. Literature was independently searched by XYW and ZX. **Supplementary Table S1** presents the literature search strategy.

### 2.2 Study selection

Inclusion criteria were: (1) Osteoporosis was diagnosed; (2) The study focused on the assessment of NLR, LMR, PLR, SII, SIRI, and other immune inflammation indexes for fracture risk prediction in the osteoporosis population; (3) Research presented odds ratios (ORs) with 95% confidence intervals (CIs), which can be accessed or computed; (4) The study population must consist of individuals aged above 18, primarily encompassing adults, postmenopausal women, and the old population; (5) Full texts were available. Exclusion criteria were: (1) Reviews, comments, meeting abstracts, case reports, as well as letters were ostracized; (2) There were duplicated or overlapping data; (3) Information for OR and 95% CI computation is insufficient.

XYW and ZX independently checked the titles and abstracts of the initially retrieved literature and downloaded and assessed publications with full texts. Dissents arising in the selection process were addressed via communication or the engagement of a third researcher (MSL).

### 2.3 Data extraction

XYW and MSL independently gathered data through Excel tables. Any dissents were settled via consensus among co-authors. The list of extracted information was developed as per the CHARMS checklist. The primary extracted study data were (16): first author, publication year, duration, country (site), sort, population, sample size, sex distribution within the study population, age, BMI, BMD, immune inflammation index, cut-off value, ORs (95% CIs) for fracture occurrence risk, standardized mean difference (SMD) for immune inflammation index value in the fracture group, and SMD for immune inflammation index value in the non-fracture cohort. Notably, for studies reporting MLR data (13, 17–19), the reciprocal of the reported ORs and their corresponding CIs was computed by reversing the values and swapping the upper and lower confidence bounds, thereby transforming MLR into LMR for better statistical analysis. In determining the cut-off value, we adopted the optimal threshold reported by the original study authors, which was derived through receiver operating characteristic (ROC) curve analysis, and subsequently extracted and evaluated the corresponding data.

### 2.4 Literature quality assessment

The Newcastle-Ottawa Scale (NOS) is a widely utilized instrument for the assessment of observational studies, applicable to both case-control and cohort designs. Accordingly, it was employed for methodological quality rating. Every study was rated in terms of selection, comparability, and outcome with the highest score of 9 (20). Studies scoring 7–9 were categorized as good quality based on previous studies that used NOS (21).

### 2.5 Statistics analysis

The pooled ORs with corresponding 95% CIs for categorical variables and SMDs for continuous variables were calculated to assess the prediction value of NLR, LMR, PLR, SII, and SIRI for the likelihood of fracture in the osteoporosis population. Correlation results for MLR were converted into the LMR format. Heterogeneity was examined via Cochran’s Q test and the Higgins I² statistic (22). *I^2^* >50% or P<0.1 denoted substantial heterogeneity. Accordingly, all analyses were carried out via a random-effects model. The robustness of the findings and possible sources of heterogeneity were investigated via sensitivity and subgroup analyses. Publication bias was detected via funnel plots and Egger’s regression tests. A two-sided P< 0.05 signified statistical significance. Our statistical analyses were enabled by STATA 18.0 and Review Manager 5.4.

## 3 Results

### 3.1 Literature screening process and results

**Figure 1:**
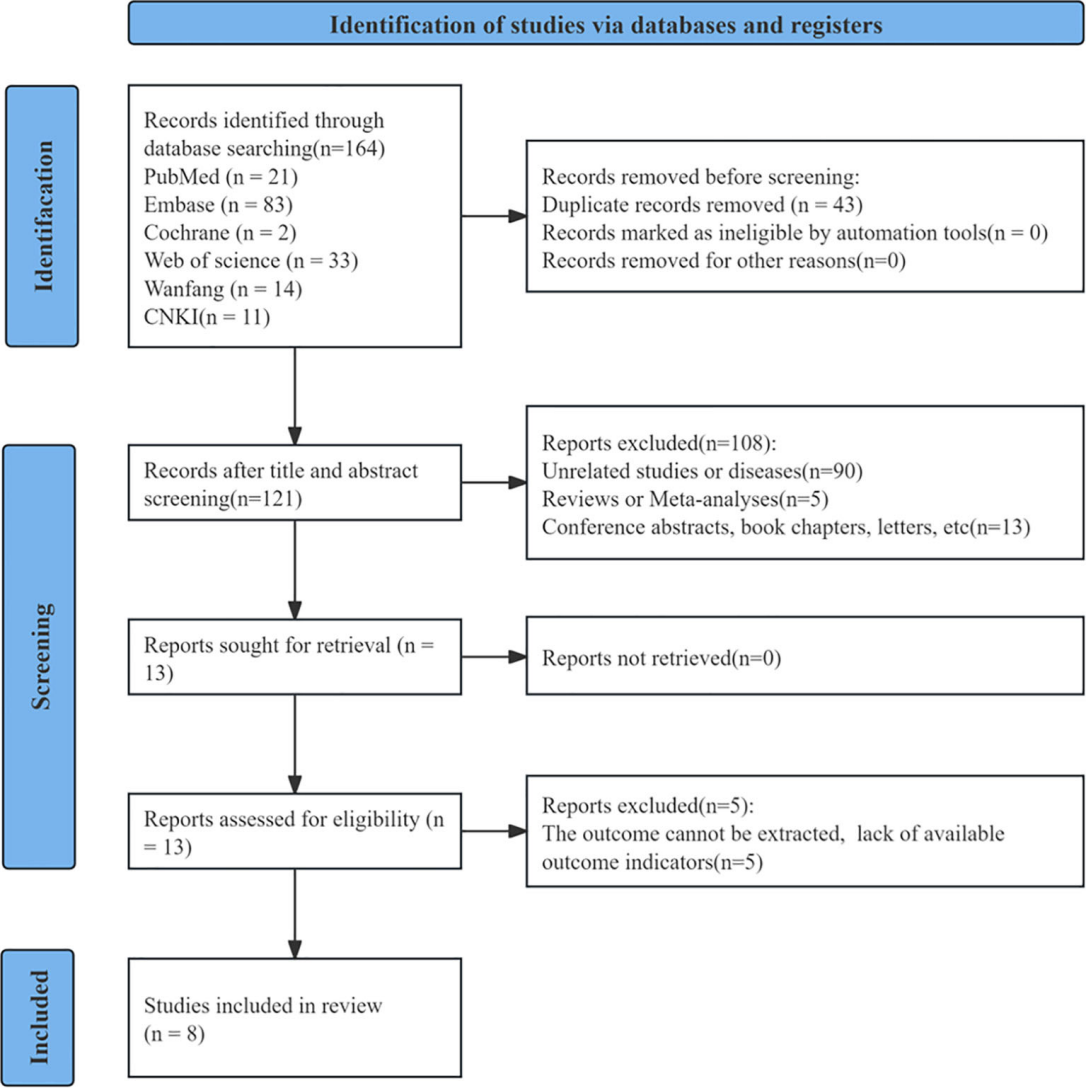
Literature retrieval process diagram.

164 papers were initially retrieved, of which 43 were eliminated owing to duplication. Upon title and abstract review of the rest of the literature, an additional 108 were removed for failing to meet the criteria on article type or for being irrelevant to the study. The investigators meticulously assessed the complete texts of the remaining 13 trials, excluding five mainly owing to insufficient pertinent data for risk analysis. At last, eight trials with 3,769 individuals were encompassed (13, 14, 17–19, 23–25). The screening process and results are displayed in **Figure 1**.

Among the eight studies identified in the literature that met the requirements, seven were executed in China, while one was carried out in South Korea. It is significant to point out that these observational researches included four cohort and four case-control studies. From 2020 to 2024, three cohort studies were published in English and one in Chinese. Among the case-control studies published from 2022 to 2024, three were in English and one in Chinese. All of them employed the immune inflammation index to assess fracture risk in individuals with osteoporosis. Notably, seven research examined the predictive significance of multiple immune inflammatory indexes. Seven analyses have demonstrated variations in the immune inflammation index values between fracture and non-fracture groups. Furthermore, five tests were mostly conducted on postmenopausal women. The characteristics of incorporated studies are presented in **Table 1**.

**Table 1:** The baseline characteristics of the included studies.

Author	Study period	Region	Study design	Population	No. of patients	Gender		Mean Age	Mean BMI	Mean BMD	Immune inflammation index	Cut-off	OR (95%CI)	Fracture Group’s Index (mean+sd+n)	Non-Fracture Group’s index(mean+sd+n)	Quality score
						Male	Female									
Li 2023a	2018-2021	China	Cohort study	Postmenopausal osteoporosis group	132	0	132	62.15 ± 6.87	22.56 ± 2.89	0.67 ± 0.09	NLR	NA	29.430(9.840-103.600)	3.77 + 0.22 + 40	3.27 + 0.22 + 92	7
Li 2023b	2018-2021	China	Cohort study	Postmenopausal osteoporosis group	132	0	132	62.15 ± 6.87	22.56 ± 2.89	0.67 ± 0.09	SII	NA	1.048(1.034-1.066)	441.32 + 29.68 + 40	426.87 + 30.57 + 92	7
Song Y 2022a	2013-2021	China	Case-control study	Osteoporosis group	426	198	228	73.85 ± 8.66	22.23 ± 4.32	NA	SII	NA	1.001(1.000-1.001)	738.93 + 145.03 + 249	548.11 + 82.49 + 177	7
Song Y 2022b	2013-2021	China	Case-control study	Osteoporosis group	426	198	228	73.85 ± 8.66	22.23 ± 4.32	NA	PLR	NA	1.008(1.005-1.011)	192.70 + 119.02 + 249	133.30 + 59.04 + 177	7
Song Y 2022c	2013-2021	China	Case-control study	Osteoporosis group	426	198	228	73.85 ± 8.66	22.23 ± 4.32	NA	NLR	NA	1.178(1.084-1.279)	3.45 + 0.61 + 249	2.55 + 0.36 + 177	7
Song Y 2022d	2013-2021	China	Case-control study	Osteoporosis group	426	198	228	73.85 ± 8.66	22.23 ± 4.32	NA	LMR	NA	0.813(0.744-0.888)	2.59 + 0.42 + 249	4.53 + 0.56 + 177	7
Chen 2024a	2022-2024	China	Case-control study	Postmenopausal osteoporosis group	658	0	658	67.5 ± 7.12	NA	NA	NLR	2.35	1.672(1.011-2.765)	2.48 + 0.90 + 169	1.77 + 0.59 + 489	6
Chen 2024b	2022-2024	China	Case-control study	Postmenopausal osteoporosis group	658	0	658	67.5 ± 7.12	NA	NA	PLR	NA	0.993(0.985-1.001)	137.31 + 37.77 + 169	121.39 + 36.04 + 489	6
Chen 2024c	2022-2024	China	Case-control study	Postmenopausal osteoporosis group	658	0	658	67.5 ± 7.12	NA	NA	SII	447.87	1.004(1.001-1.006)	537.56 + 227.83 + 169	393.98 + 150.71 + 489	6
Chen 2024d	2022-2024	China	Case-control study	Postmenopausal osteoporosis group	658	0	658	67.5 ± 7.12	NA	NA	LMR	NA	0.919(0.866-0.950)	3.70 + 1.25 + 169	4.55 + 1.24 + 489	6
Zhang 2024	2018-2023	China	Case-control study	Postmenopausal osteoporosis group	370	0	370	70.61 ± 11.40	22.47 ± 3.25	NA	NLR	NA	2.163(1.105-4.237)	3.27 + 2.09 + 256	1.83 + 1.12 + 114	7
Fang 2020a	2015-2017	China	Cohort study	Postmenopausal osteoporosis group	238	0	238	60.5 ± 8.1	23.4 ± 1.1	0.71 ± 0.12	NLR	3.64	2.11 (1.37-3.25)	3.29 + 2.06 + 92	2.68 + 1.70 + 146	7
Fang 2020b	2015-2017	China	Cohort study	Postmenopausal osteoporosis group	238	0	238	60.5 ± 8.1	23.4 ± 1.1	0.71 ± 0.12	SII	834.89	3.02 (1.98-4.61)	766.2 + 562.1 + 92	604.3 + 448.90 + 146	7
Fang 2020c	2015-2017	China	Cohort study	Postmenopausal osteoporosis group	238	0	238	60.5 ± 8.1	23.4 ± 1.1	0.71 ± 0.12	PLR	161.94	1.80 (1.06-3.07)	146.5 + 78.0 + 92	135.8 + 67.10 + 146	7
Fang 2020d	2015-2017	China	Cohort study	Postmenopausal osteoporosis group	238	0	238	60.5 ± 8.1	23.4 ± 1.1	0.71 ± 0.12	LMR	4.16	0.44 (0.26-0.75)	3.46 + 1.66 + 92	3.95 + 1.60 + 146	7
Liu 2024a	2011-2023	China	Cohort study	Osteoporosis group	1222	1128	94	61.72 ± 8.92	23.22 ± 3.41	0.941 ± 0.128	NLR	NA	1.247 (1.189-1.309)	2.57 + 1.77 + 206	1.66 + 0.68 + 1016	7
Liu 2024b	2011-2023	China	Cohort study	Osteoporosis group	1222	1128	94	61.72 ± 8.92	23.22 ± 3.41	0.941 ± 0.128	PLR	NA	1.398 (1.251-1.563)	141.98 + 80.08 + 206	112.35 + 41.73 + 1016	7
Liu 2024c	2011-2023	China	Cohort study	Osteoporosis group	1222	1128	94	61.72 ± 8.92	23.22 ± 3.41	0.941 ± 0.128	LMR	NA	0.838 (0.775-0.905)	4.00 + 1.96 + 206	5.56 + 2.07 + 1016	7
Liu 2024d	2011-2023	China	Cohort study	Osteoporosis group	1222	1128	94	61.72 ± 8.92	23.22 ± 3.41	0.941 ± 0.128	SII	NA	1.092 (1.012-1.178)	552.32 + 474.18 + 206	367.81 + 182.20 + 1016	7
Liu 2024e	2011-2023	China	Cohort study	Osteoporosis group	1222	1128	94	61.72 ± 8.92	23.22 ± 3.41	0.941 ± 0.128	SIRI	NA	1.062 (1.009-1.117)	1.04 + 0.78 + 206	0.61 + 0.36 + 1016	7
Fu 2024a	2020-2023	China	Case-control study	Osteoporosis group	310	37	273	67.38 ± 9.62	21.91 ± 3.12	0.569 ± 0.096	NLR	2.307	1.480 (1.114-1.966)	2.32 + 1.48 + 135	1.97 + 1.02 + 175	7
Fu 2024b	2020-2023	China	Case-control study	Osteoporosis group	310	37	273	67.38 ± 9.62	21.91 ± 3.12	0.569 ± 0.096	LMR	0.201	0.954 (0.920-0.989)	4.35 + 2.12 + 135	5.56 + 4.60 + 175	7
Fu 2024c	2020-2023	China	Case-control study	Osteoporosis group	310	37	273	67.38 ± 9.62	21.91 ± 3.12	0.569 ± 0.096	SII	541.894	1.001(1.000-1.002)	432.73 + 295.65 + 135	377.21 + 208.97 + 175	7
Fu 2024d	2020-2023	China	Case-control study	Osteoporosis group	310	37	273	67.38 ± 9.62	21.91 ± 3.12	0.569 ± 0.096	SIRI	0.632	3.327(1.510-7.330)	0.69 + 0.44 + 135	0.50 + 0.43 + 175	7
Song 2022a	2005-2017	Korea	Cohort study	Postmenopausal osteoporosis group	413	0	413	61.9 ± 8.6	22.8 ± 2.8	0.82 ± 0.13	NLR	NA	4.72(2.27-9.83)	NA	NA	6
Song 2022b	2005-2017	Korea	Cohort study	Postmenopausal osteoporosis group	413	0	413	61.9 ± 8.6	22.8 ± 2.8	0.82 ± 0.13	PLR	NA	1.96(1.09-3.53)	NA	NA	6
Song 2022c	2005-2017	Korea	Cohort study	Postmenopausal osteoporosis group	413	0	413	61.9 ± 8.6	22.8 ± 2.8	0.82 ± 0.13	LMR	NA	0.379(0.204-0.699)	NA	NA	6

NLR, neutrophil/lymphocyte ratio; LMR, lymphocyte/monocyte ratio; PLR, platelet/lymphocyte ratio;SII, systemic immune inflammation index; SIRI, systemic inflammation response index; NA, not available.

### 3.2 Study quality

All studies scored 6–7 in NOS, with six studies being of high quality (**Supplementary Table S2**).

### 3.3 Meta-analysis results

#### 3.3.1 Predictive value of NLR

**Figure 2:**
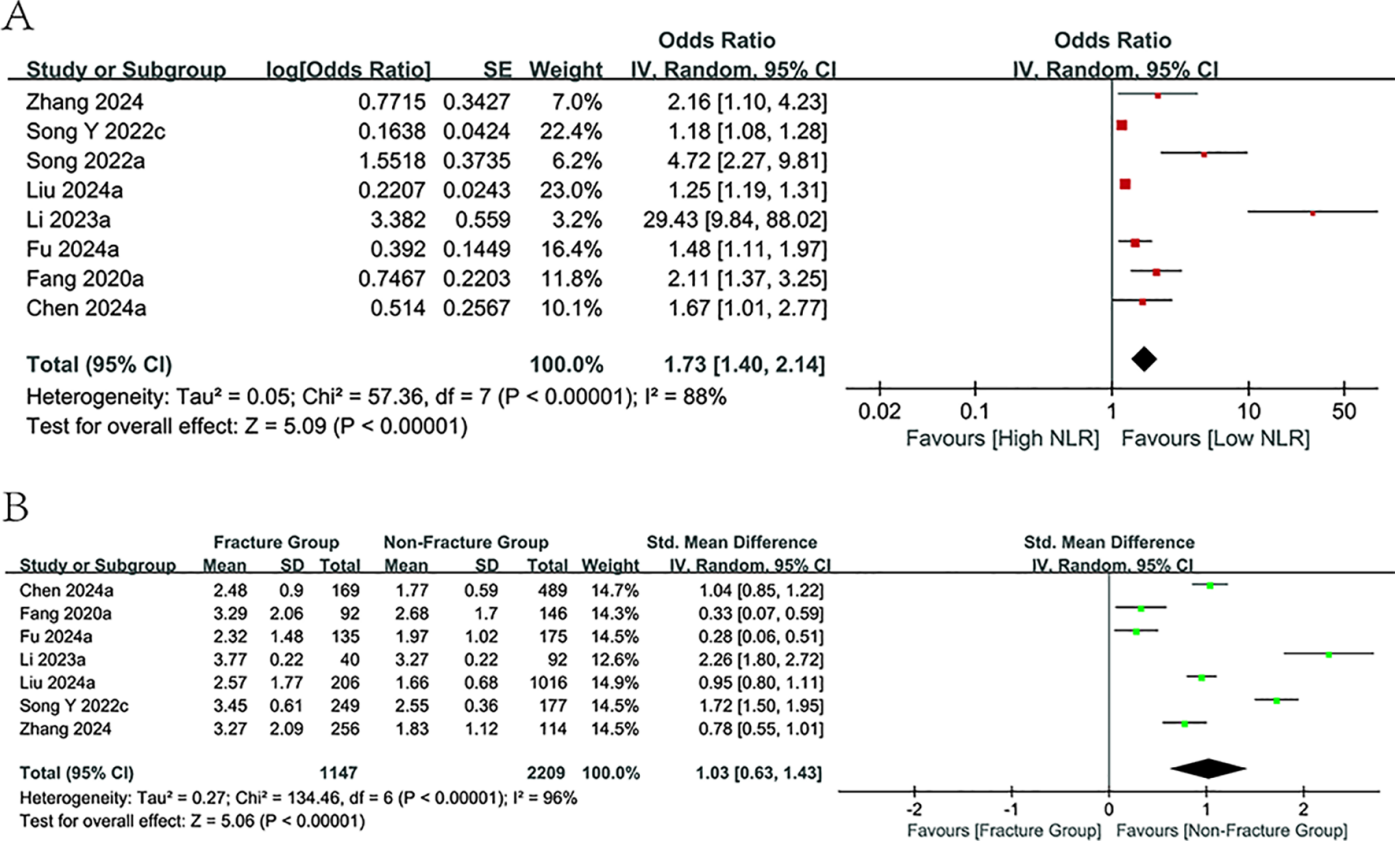
Forest plots for the association between NLR and fracture risk.

The prognosis value of NLR for fracture prediction among the osteoporotic population was evaluated across eight observational studies involving 3,769 patients. All included studies reported relevant ORs and 95% CIs, with seven additionally providing NLR values stratified by fracture status within the osteoporotic cohort. Considerable heterogeneity (*I^2^* = 88%, p<0.00001; *I^2^* = 96%, p<0.00001) prompted the adoption of a random-effects model (**Figure 2**). Our meta-analysis demonstrated a notable relation of elevated NLR values to a rising fracture risk (OR = 1.73, 95% CI: 1.40-2.14; p < 0.00001, **Figure 2A**). Furthermore, NLR levels were markedly higher in the fracture cohort in comparison to the non-fracture cohort (SMD = 1.03, 95% CI: 0.63-1.43; p < 0.00001, **Figure 2B**). Subgroup analyses by design, mean age, and population were carried out (**Table 2**). The predictive value of NLR was consistently observed across all subgroups, with a substantial reduction in heterogeneity noted within the subgroups defined by the study population (*I²* = 32%).

**Table 2:** Pooled ORs for NLR, LMR, SII and PLR in subgroup analyses.

Subgroup	NLR				LMR				SII				PLR			
	Study group	OR [95%CI]	P value	*I^2^*	Study group	OR [95%CI]	P value	*I^2^*	Study group	OR [95%CI]	P value	*I^2^*	Study group	OR [95%CI]	P value	*I^2^*
*Total*	8	1.73 [1.40, 2.14]	<0.00001	88%	6	0.85 [0.77, 0.93]	0.0007	84%	6	1.01 [1.00, 1.01]	0.008	94%	5	1.03 [1.00, 1.06]	0.10	NA
Study design																
Cohort study	4	3.77 [1.51, 9.44]	0.005	94%	3	0.55 [0.31, 0.98]	0.04	83%	3	1.22 [1.02, 1.45]	0.03	92%	3	1.43 [1.28, 1.59]	<0.00001	0%
Case-control study	4	1.41 [1.11, 1.78]	0.004	56%	3	0.90 [0.83, 0.98]	0.01	82%	3	1.00 [1.00, 1.00]	0.01	47%	2	1.00 [0.99, 1.02]	0.9	NA
Mean age																
≥65year	4	1.41 [1.11, 1.78]	0.004	56%	3	0.90 [0.83, 0.98]	0.01	82%	3	1.00 [1.00, 1.00]	0.01	47%	2	1.00 [0.99, 1.02]	0.9	NA
<65year	4	3.77 [1.51, 9.44]	0.005	94%	3	0.55 [0.31, 0.98]	0.04	83%	3	1.22 [1.02, 1.45]	0.03	92%	3	1.43 [1.28, 1.59]	<0.00001	0%
Study Population																
Postmenopausal osteoporosis group	5	3.56 [1.74, 7.25]	0.0005	84%	3	0.56 [0.29, 1.08]	0.08	NA	3	1.04 [0.99, 1.10]	0.13	NA	3	1.43 [0.85, 2.39]	0.18	NA
Unclassified osteoporosis population group	3	1.23 [1.16, 1.31]	<0.00001	32%	3	0.87 [0.78, 0.97]	0.01	88%	3	1.00 [1.00, 1.00]	0.15	NA	2	1.18 [0.86, 1.63]	0.31	NA

NLR, neutrophil/lymphocyte ratio; LMR, lymphocyte/monocyte ratio; PLR, platelet/lymphocyte ratio;SII, systemic immune inflammation index; SIRI, systemic inflammation response index; NA, not available. The unclassified osteoporosis group refers to patients with primary osteoporosis who have not been explicitly categorized as having either senile osteoporosis or postmenopausal osteoporosis.

#### 3.3.2 Predictive value of LMR

**Figure 3:**
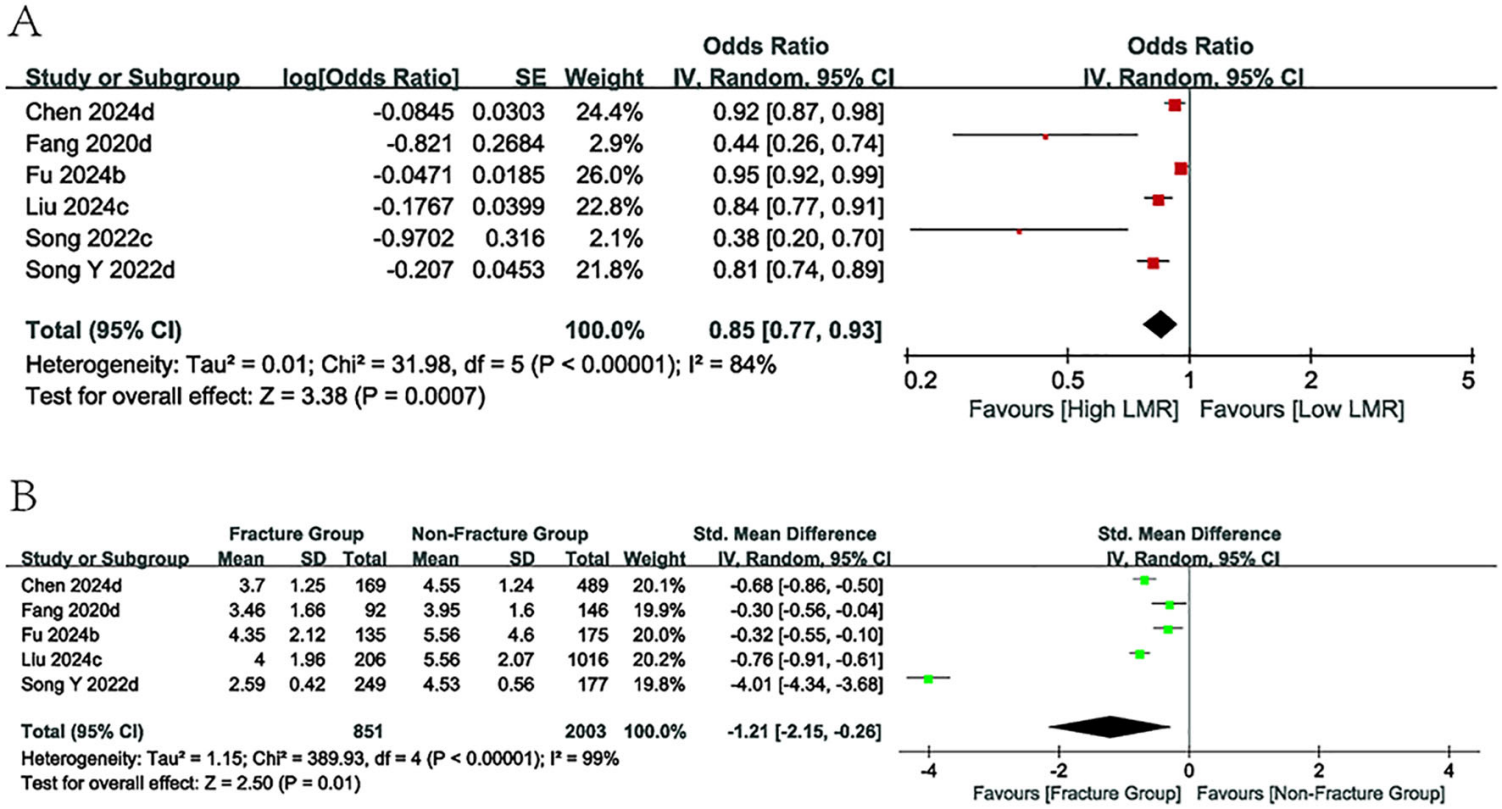
Forest plots for the association between LMR and fracture risk.

Six studies reported ORs and 95% CIs for LMR levels. Among these, five studies provided LMR values for both the fracture and non-fracture groups. The pooled analysis demonstrated a significant inverse association between LMR levels and fracture risk in individuals with osteoporosis (OR = 0.85, 95% CI: 0.77–0.93; p = 0.0007; **Figure 3A**). Moreover, patients in the fracture group exhibited markedly lower LMR levels compared with those in the non-fracture group (SMD = -1.21, 95% CI: -2.15 to -0.26; p = 0.01; **Figure 3B**). Subgroup analyses indicated that no significant association was observed between LMR levels and fracture risk in the postmenopausal osteoporosis population, whereas correlations were evident in other subgroups.

#### 3.3.3 Predictive value of SII

**Figure 4:**
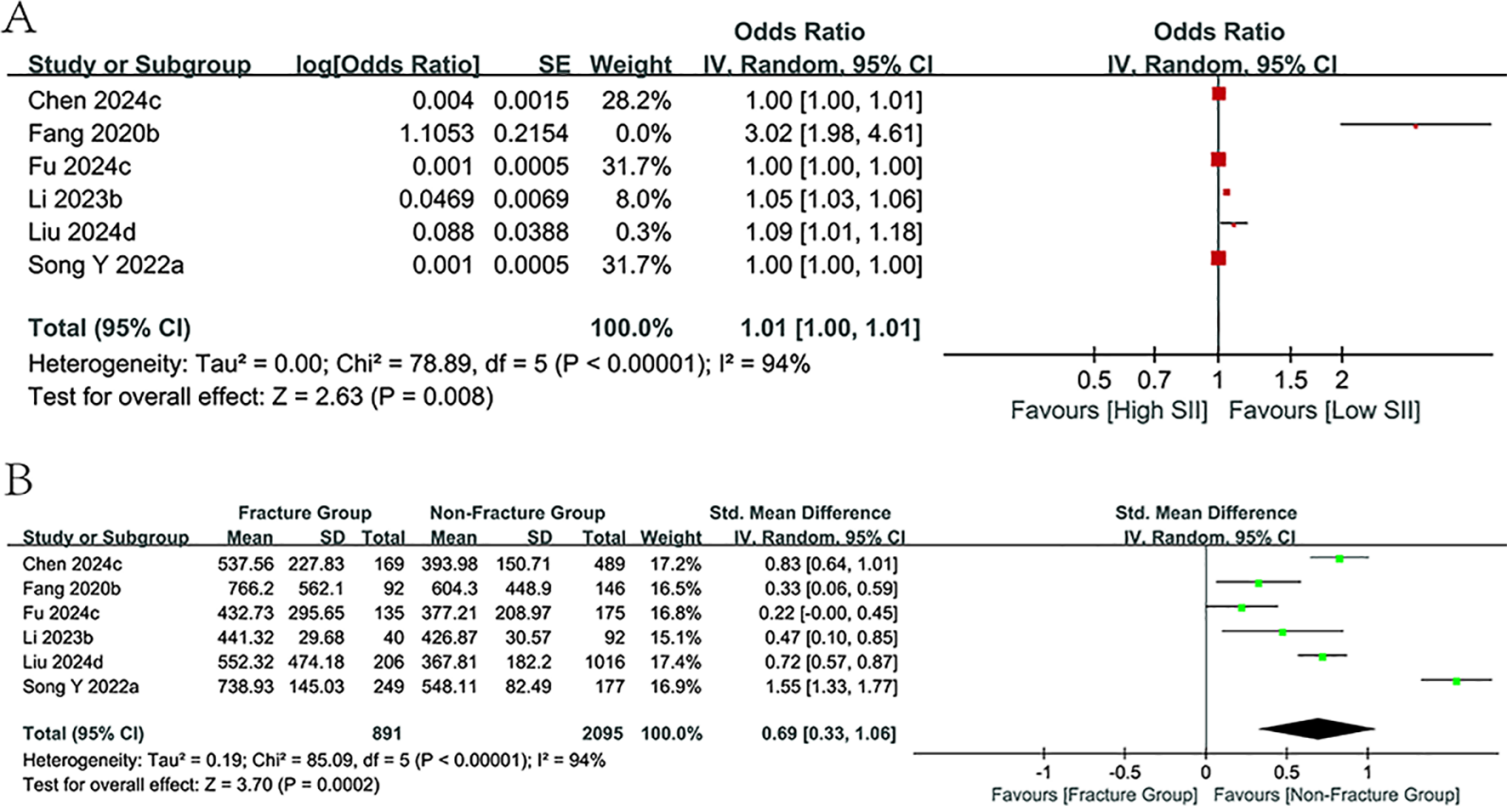
Forest plots for the association between SII and fracture risk.

Six articles provided data on ORs, 95% Cls, and comparisons between fracture and non-fracture groups related to SII levels. The pooled results from the literature suggested that the SII level was positively connected with the likelihood of fracture (OR = 1.01, 95% CI: 1.00-1.01; p=0.008, **Figure 4A**), with the fracture group exhibiting greater SII values than the non-fracture group (SMD = 0.69, 95% CI:0.33-1.06; p=0.0002, **Figure 4B**). Subgroup analyses confirmed the use of SII values in forecasting fracture incidence across all subgroups, with the exception of the study population subgroup. Meanwhile, among the subgroups of the case-control research and those aged 65 years and older, an obvious decrease in heterogeneity is observed (*I^2^* = 47%).

#### 3.3.4 Predictive value of PLR

**Figure 5:**
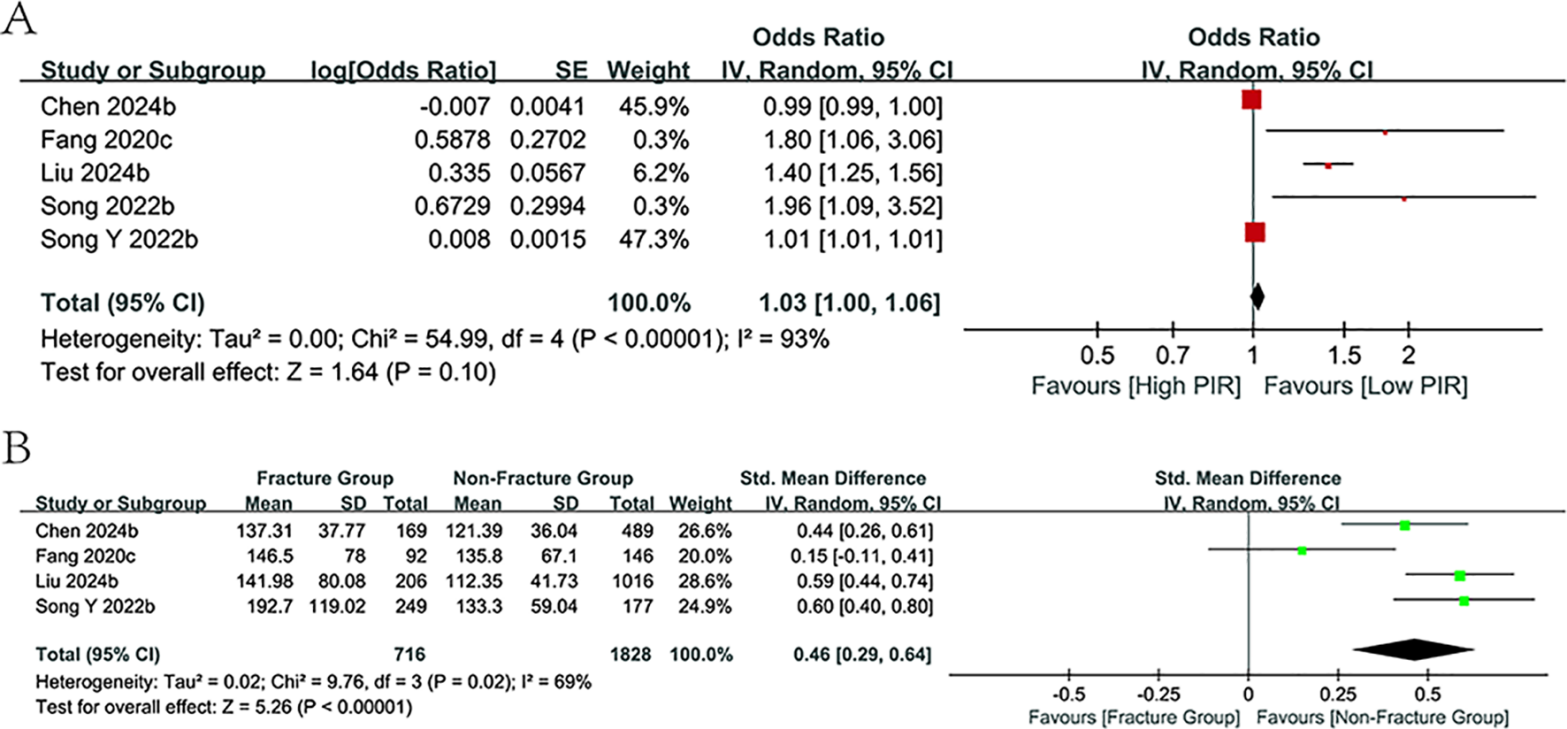
Forest plots for the association between PLR and fracture risk.

Five investigations examined the correlation between PLR values and the likelihood of fractures in osteoporosis patients, with four studies presenting PLR values for both fracture and non-fracture groups. The study found no link between PLR levels with fracture risk (p=0.10, **Figure 5A**); however, PLR values were markedly higher in the fracture cohort than in the non-fracture cohort (SMD = 0.46, 95% CI:0.29-0.64; p<0.00001, **Figure 5B**). Further subgroup analyses proved the predictive relevance of PLR values within the cohort study subgroups and those with a mean age under 65 years (*I^2^* = 0%). However, no association was observed in the other subgroups.

#### 3.3.5 Predictive value of SIRI

**Figure 6:**
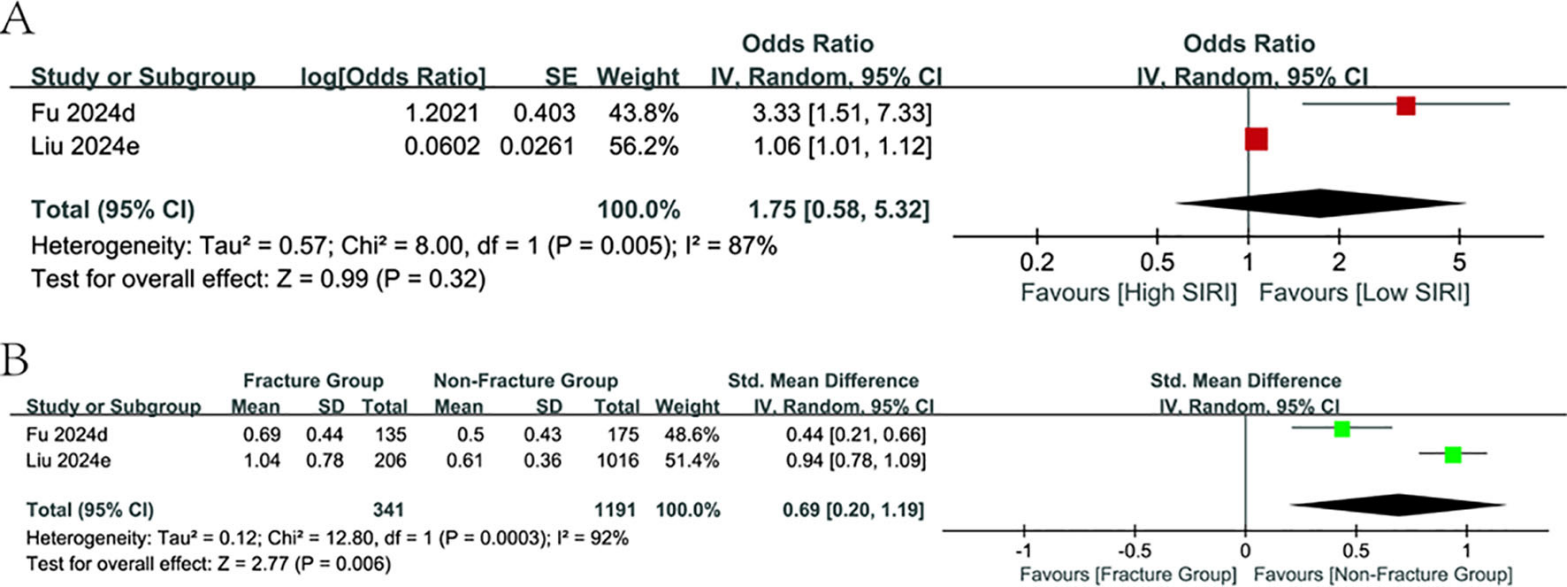
Forest plots for the association between SIRI and fracture risk.

Only two publications have carried out assessments focusing on SIRI values in the osteoporosis population, covering 1,532 people. The meta-analysis findings concluded that the SIRI values, which were heightened in the fracture group relative to the non-fracture group (SMD = 0.69, 95% CI:0.20-1.19; p=0.006, **Figure 6B**), were not correlated with fracture risk or had predictive relevance in the study of categorical variables (p=0.32, **Figure 6A**).

### 3.4 Sensitivity analysis

**Figure 7:**
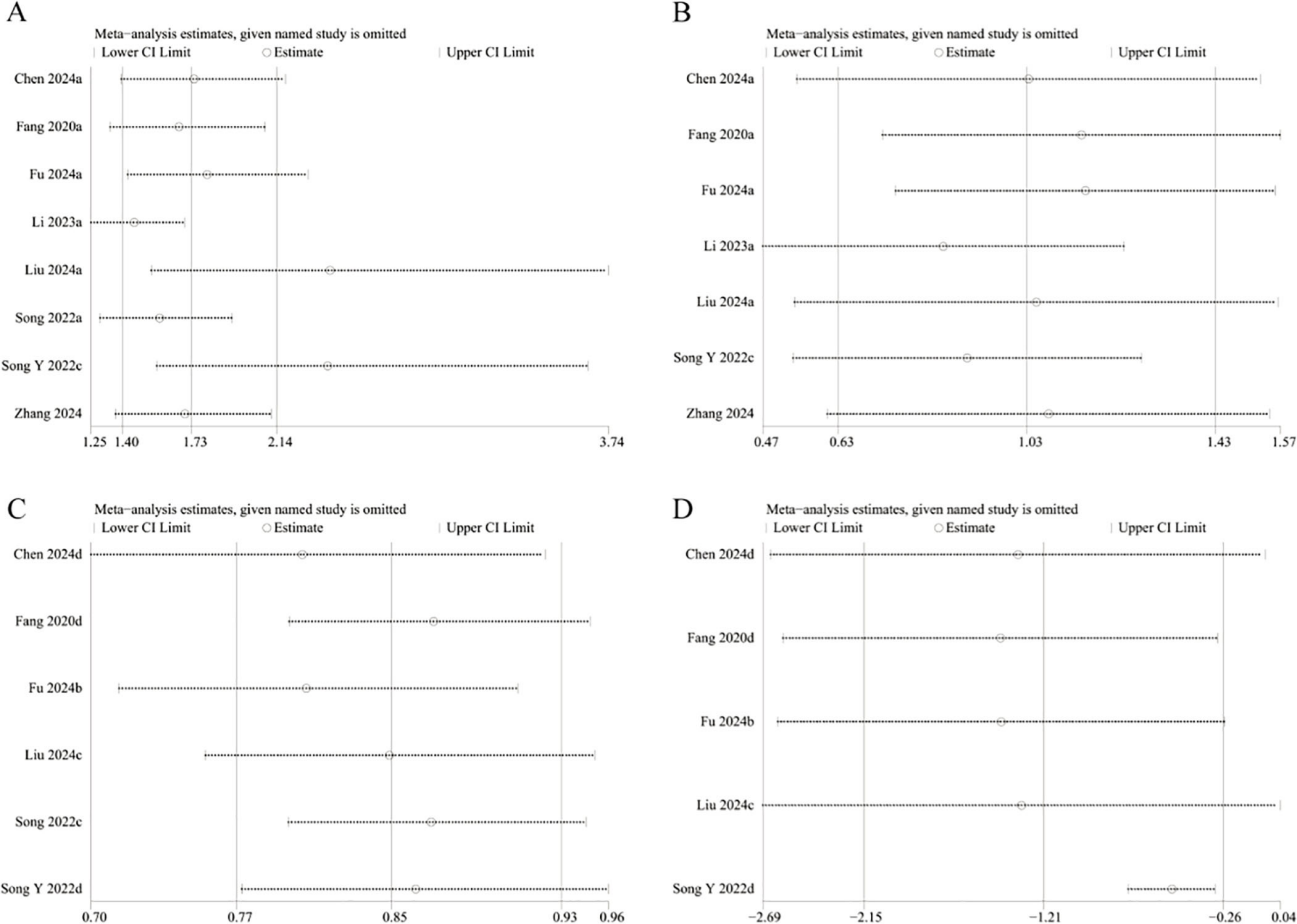
Sensitivity analysis of NLR (**A**: categorical variable; **B**: continuous variable) and LMR (**C**: categorical variable; **D**: continuous variable).

**Figure 8:**
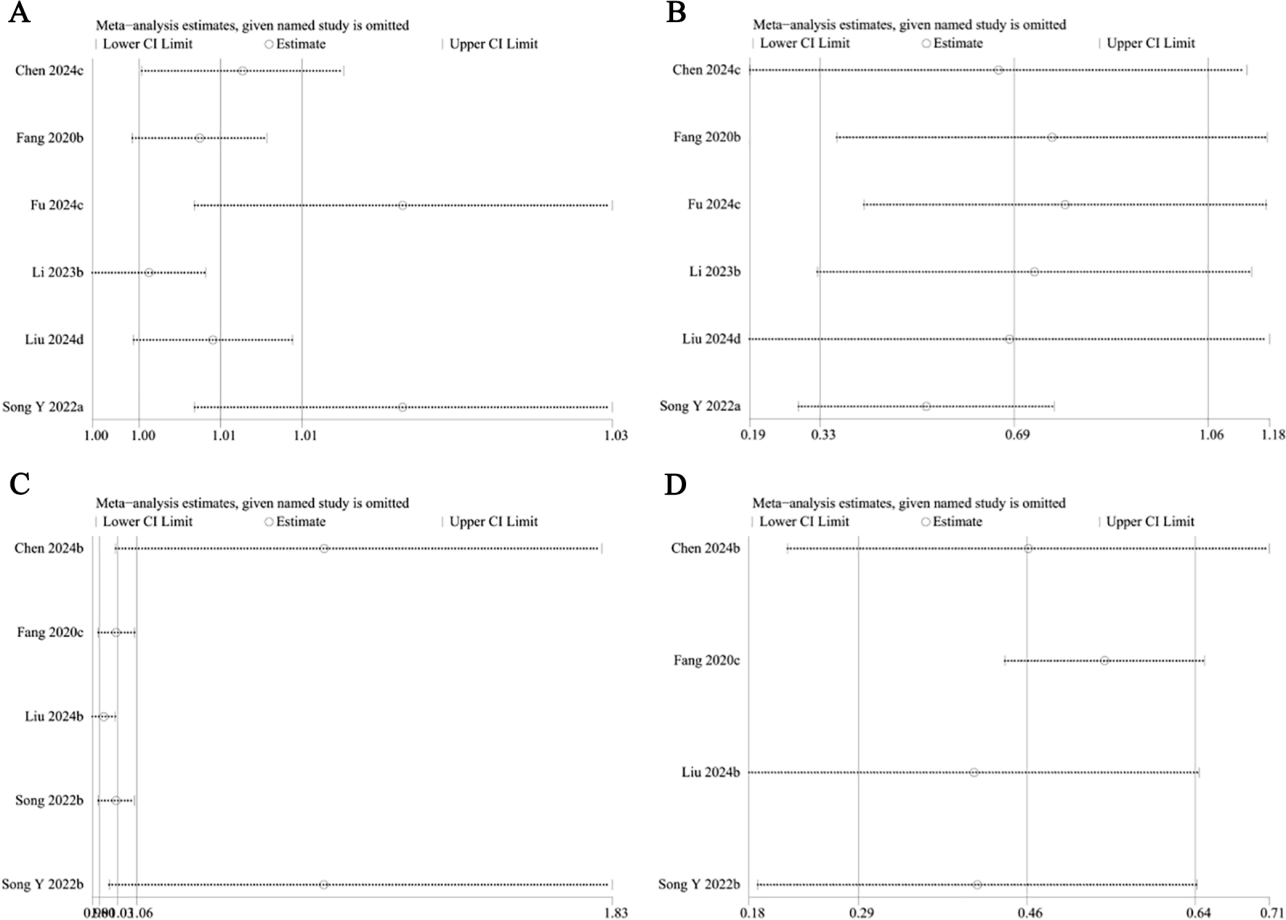
Sensitivity analysis of SII (**A**: categorical variable; **B**: continuous variable) and PLR (**C**: categorical variable; **D**: continuous variable).

The results’ robustness and the clinical importance of the different immune inflammation indexes were evaluated via sensitivity analyses. The findings regarding NLR demonstrated that with each study being systematically omitted, the effect sizes continued to fall within the initial range (**Figures 7A, B**). This indicated no individual study exerted a disproportionate influence on the link of NLR to fracture risk, thereby revealing the robustness of our analysis. However, the sensitivity analysis of the continuous variable study regarding LMR revealed that the effect sizes deviated from the initial range upon removal of Liu et al.’s study (18) (**Figure 7D**). This suggests that the model exhibits significant instability. Sensitivity analysis regarding the categorical variable study on SII revealed that effect sizes diminished beyond the initial range following the deletion of Li et al. study (23) (**Figure 8A**). It means that the study influenced the predictive capacity of SII values concerning fracture risk, and the consistency of the results needs further improvement. The analysis of the categorical variable study on PLR demonstrated high model sensitivity (**Figure 8C**). The effect sizes underwent considerable alterations and exceeded the initial range following the successive exclusion of the investigations by Fang et al. (14), Liu et al. (18), and Song et al. (19). However, sensitivity analysis for SIRI could not be performed due to an insufficient number of available studies (fewer than three).

### 3.5 Publication bias

**Figure 9:**
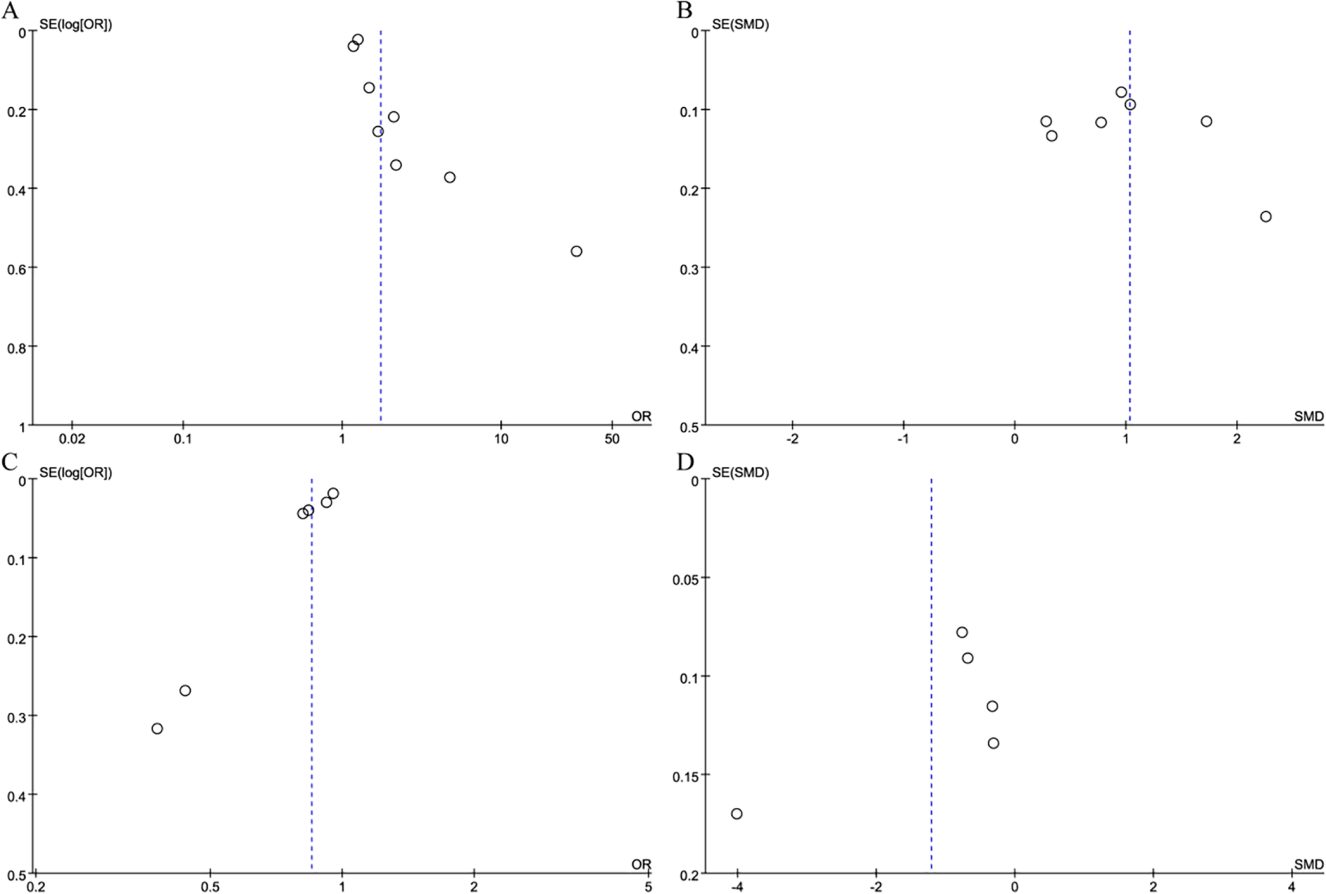
Funnel plot for the evaluation of publication bias for NLR (**A**: categorical variable; **B**: continuous variable) and LMR (**C**: categorical variable; **D**: continuous variable).

**Figure 10:**
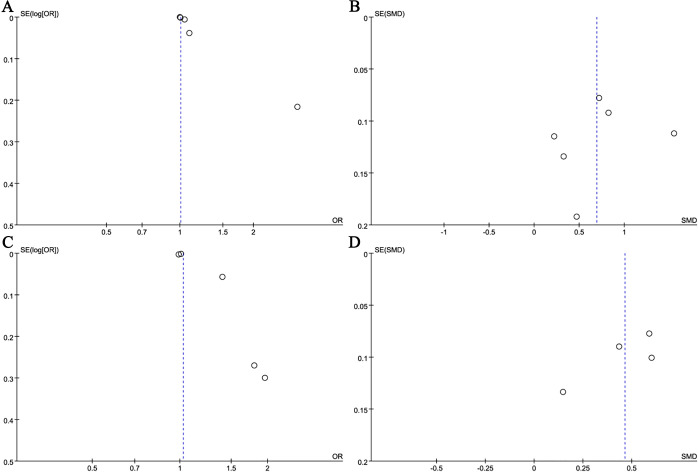
Funnel plot for the evaluation of publication bias for SII (**A**: categorical variable; **B**: continuous variable) and PLR (**C**: categorical variable; **D**: continuous variable).

Publication bias was found via funnel plots and Egger’s tests. Firstly, funnel plots indicated publication bias for NLR (continuous variable, **Figure 9B**), LMR (categorical variable, **Figure 9C**), LMR (continuous variable, **Figure 9D**) and SII (categorical variable, **Figure 10A**), whereas no publication bias was observed for NLR (categorical variable, **Figure 9A**), SII (continuous variable, **Figure 10B**), PLR (categorical variable, **Figure 10C**), and PLR (continuous variable, **Figure 10D**). Furthermore, Egger’s test corroborated the existence of publishing bias for NLR (categorical variable, P = 0.012), LMR (categorical variable, P = 0.005), and SII (categorical variable, P = 0.01), while indicating an absence of publication bias for the other indicators. Moreover, the limited research on SIRI precluded us from conducting a publishing bias study (<3 studies). **Table 3** presents a summary of the key findings derived from the sensitivity and publication bias analyses.

**Table 3:** Summary of sensitivity analysis and publication bias results.

Immune inflammation index	Number of included studies	Sensitivity analysis results	Egger’s test P value
NLR^a^	8	Stable	0.012
NLR^b^	7	Stable	0.621
LMR^a^	6	Stable	0.005
LMR^b^	5	The results are unstable, with the instability originating from the study by Liu et al. (18).	0.320
SII^a^	6	The results are unstable, with the instability originating from the study by Li et al. (23).	0.010
SII^b^	6	Stable	0.676
PLR^a^	5	The results are unstable, with the instability originating from the study by Fang et al. (14), Liu et al. (18), and Song et al. (19).	0.294
PLR^b^	4	Stable	0.201
SIRI^a^	2	NA	NA
SIRI^b^	2	NA	NA

NLR, neutrophil/lymphocyte ratio; LMR, lymphocyte/monocyte ratio; PLR, platelet/lymphocyte ratio;SII, systemic immune inflammation index; SIRI, systemic inflammation response index; a, categorical variable; b, continuous variable; NA, not available.

## 4 Discussion

An imbalance in bone metabolism may give rise to a range of metabolic bone disorders like osteoporosis (26, 27). Immune dysregulation and the activation of systemic inflammatory responses are pivotal in osteoporosis and fragility fractures. The inflammatory processes and immune disturbances disrupt bone homeostasis, promote osteoclastogenesis, and enhance osteoclast activity, thereby lowering bone mineral density and strength, ultimately markedly raising the likelihood of fracture in individuals with osteoporosis (10, 28, 29). Research on postmenopausal osteoporosis indicates that menopause induces alterations in the immunological and inflammatory milieu due to age, calcium depletion, and diminished estrogen levels, which profoundly impact the microscopic architecture of bones. Specifically, inflammatory cells within the bone marrow stimulate cytokine and chemokine secretion, causing osteoporosis and elevating the fracture risk in females (14, 30). In recent years, a variety of inflammation-related disorders like chronic obstructive pulmonary disease, ankylosing spondylitis, systemic lupus erythematosus, as well as osteoarthritis were identified as risk factors for osteoporosis and fragility fractures (31–33). Inflammation is now recognized not only as a central pathogenic mechanism in the progression of osteoporosis but also as an important predictor of disease onset and fracture risk (13). Plenty of biomarkers obtained from normal blood testing, including NLR, LMR, SII, PLR, and SIRI, can indicate systemic inflammation and immunological state. Given their accessibility, cost-effectiveness, and comprehensiveness, these routinely available blood-based markers hold promise for predicting both osteoporosis and fracture risk, warranting further exploration. These indexes have already demonstrated diagnostic and prognostic utility in oncological and autoimmune diseases (21). Building upon this theoretical foundation, our study seeks to first evaluate whether multiple immune inflammatory indexes reliably predict the fracture risk among the osteoporosis population, thereby unveiling how inflammation influences bone health and informing future research in this domain.

This meta-analysis encompassed 3,769 patients and endeavored to evaluate the predictive relevance of the immune inflammation index (NLR, LMR, SII, PLR, SIRI) regarding the probability of fracture incidence in osteoporotic individuals. Our research has demonstrated that NLR, LMR, and SII have considerable predictive value for fracture risk in osteoporotic individuals. Meanwhile, the results of categorical and continuous variables for NLR, LMR, and SII between fracture and non-fracture groups were statistically significant, corroborating the strong correlation between the three indexes and the likelihood of fracture occurrence. Although the PLR and SIRI demonstrated notable disparities between the fracture and non-fracture groups, a consistent association was not observed when analyzed as categorical variables. The sensitivity analysis demonstrated that the pooled effect estimates for several indices, including NLR, LMR, SII, and PLR, exhibited notable fluctuations when individual studies were sequentially excluded, suggesting a certain degree of instability in the aggregated data. Such instability may stem from the predominantly retrospective nature of the included studies, which introduces potential uncontrolled confounding biases (14, 19, 23); inconsistencies in the timing of immune inflammatory index assessments and the lack of standardized cut-off definitions across studies; as well as the limited sample sizes in some investigations, particularly regarding fracture event counts, which may result in insufficient statistical power. Furthermore, heterogeneity in the study populations (for example, differences in menopausal status) may compromise the reliability of the estimated associations. Accordingly, future studies should aim to standardize both the measurement protocols and reporting criteria for these indices, and employ more sophisticated multivariable adjustment models in statistical analyses to enhance the robustness and generalizability of the findings. The assessment of publication bias revealed evidence of bias for the predictive indices NLR, LMR, and SII in categorical analyses. This phenomenon may be attributable to the retrospective design of the included studies and their geographic concentration within Asian populations. Therefore, the strength of associations reported in the present study should be interpreted with caution, as they may reflect a combination of genuine effects and publication bias. Ensuring research integrity in this field will require the inclusion of unpublished negative results and the conduct of prospective investigations to achieve a more balanced and objective risk estimation.

In the analysis, three primary subgroups, study design, mean age, and study population were established to evaluate the study content and to examine the correlation between various immune inflammatory indexes and the risk of fracture development. It is important to note that the “unclassified osteoporosis” group refers to patients with primary osteoporosis who have not been clearly categorized as having either senile or postmenopausal osteoporosis. The investigation revealed a decrease in sensitivity to immune inflammation indexes among patients aged 65 and older. This diminished responsiveness may be attributed to the age-related decline in innate immune function, commonly referred to as immunosenescence, which impairs the capacity of older individuals to mount effective physiological responses to fracture-induced stress, thereby reducing the prognostic utility of these biomarkers (34, 35). Moreover, within the NLR subgroup, studies targeting specific postmenopausal osteoporosis populations demonstrated a higher sensitivity to disease incidence compared to those that did not stratify osteoporosis populations. The foregoing findings reflect the imperative for future research to prioritize osteoporosis patient stratification by sex and to conduct independent investigations into the dynamic changes in inflammatory and immunological status before and after fracture events. Additionally, the substantial heterogeneity observed in the subgroup analyses may be attributed to multiple factors. In addition to variations in study design and population characteristics, clinical differences, such as disparities in fracture outcomes (e.g., vertebral versus hip fractures) across studies, may have introduced confounding effects by directly influencing systemic inflammatory responses. Moreover, inconsistencies in osteoporosis treatment protocols among study groups may have masked the true impact of immune inflammation indexes. From a methodological perspective, variability in blood sample analyzers and differences in the timing of biomarker assessments could have further contributed to measurement inconsistencies. Collectively, these factors account for the observed variability in outcomes. Future studies should emphasize the use of comprehensive and standardized testing methodologies to better control for potential confounding variables.

In the skeletal system, chronic inflammation and immune system activation directly influence bone integrity and quality. Evidence from studies on postmenopausal osteoporosis suggests that estrogen deficiency induces a persistent pro-inflammatory state, significantly increasing the secretion of inflammatory mediators within the local microenvironment, including interleukins (ILs), tumor necrosis factor-alpha (TNF-α), IL-1β, as well as reactive oxygen species (ROS) (12, 36, 37). These mediators influence the equilibrium of bone metabolism, facilitating the onset of osteoporosis and fragility fractures. Innate immune cells are the main sources of pro-inflammation mediators (37). Macrophages, derived from the monocyte lineage, exhibit pro-inflammatory (M1) and anti-inflammatory (M2) phenotypes based on the immunological milieu (38). Their considerable plasticity allows them to shift between M1 and M2 states in response to environmental cues (39). M1 macrophages serve as precursors to osteoclasts. Prior investigations have demonstrated that stimulation of macrophages with established M1 inducers leads to a downregulation of osteoinductive factors like morphogenetic proteins BMP-2 and BMP-6 (38). Another study involving ovariectomized osteoporotic C57BL/6 mice proved that M1 and M2 in the bone marrow differentiate into functional osteoclasts under the influence of RANKL, with M2 macrophages contributing more significantly to this differentiation. This process ultimately resulted in an elevated M1/M2 ratio (40). Neutrophils, produced by precursors in the bone marrow, participate in innate immune responses and are implicated in bone loss via chemokine secretion and osteoclastogenic cell recruitment (41). Previous research has demonstrated that neutrophils play a pivotal role in the pathogenesis of inflammatory diseases. Their involvement encompasses the secretion of proteolytic enzymes, the formation of neutrophil extracellular traps (NETs), and the modulation of other immune cell activities (42).

Additionally, evidence indicates a robust link between the elevation of neutrophils induced by RANKL and reduced bone density in inflammatory conditions (43). Monocytes act as progenitors to osteoclasts and modulate inflammatory responses by secreting cytokines like IL-1, TNF, CXCL10, as well as IL-15, thereby facilitating osteoclast-mediated bone resorption and bone metabolism regulation (37, 44)]. Lymphocytes, including T and B cells, interact with osteoblasts and osteoclasts both directly and indirectly across cells, thereby influencing bone homeostasis. T cells demonstrate opposing roles in bone remodeling; while quiescent Txcells confer protective effects by limiting osteoclast activity, activated T cells promote osteoclastogenesis (45). In chronic inflammatory situations, regulatory T cells (Tregs) inhibit the activity of helper T cells 17 (Th17) (45). Additionally, there is an increase in inflammatory cytokines, including NFATc1, TNF-α, and IL-1β, synthesized by T cells (46). These methods render T lymphocytes pivotal in inflammation-induced bone resorption. B cells contribute to bone control akin to T cells by generating bone marrow-derived OPG under physiological conditions to suppress osteoclast development. Findings show that B cells, triggered by estrogen deficiency and inflammatory states, facilitate bone resorption via G-CSF and RANKL production (47). Under conditions of inflammatory activation, platelets directly interact with circulating leukocytes, resulting in alterations in the surface expression of P-selectin and CD40L. Moreover, platelets form specialized aggregates with monocytes and neutrophils, thereby participating actively in immune and inflammatory responses (48, 49). A cross-sectional study by Zhang et al. demonstrated a negative association between platelet count and BMD, underscoring the necessity of continuous platelet count monitoring in patients with osteoporosis (50). In conclusion, this inflammatory milieu facilitates the activation and recruitment of multiple blood cell types, including neutrophils, monocytes, and platelets. Such phenomena are reflected by variations in immune inflammation indexes. The intricate mechanisms linking immune-mediated inflammation and osteoporosis require further investigation. The intercellular interactions involved remain incompletely characterized. The present findings are consistent with prior evidence, demonstrating associations between alterations in several immune inflammatory indexes and increased risk of osteoporosis and related fractures.

Despite its clinical utility, the present meta-analysis is subject to several limitations that warrant consideration. A notable limitation of the present study lies in the fact that all included literature originated from East Asia (predominantly China), thereby restricting the generalizability of the findings across different racial groups. Variations in bone metabolic characteristics, genetic backgrounds, and comorbidity profiles among diverse populations may substantially influence both the strength of associations and the optimal cut-off values between immune inflammation indexes and fracture risk. Consequently, caution should be exercised when extrapolating these results to non-Asian populations. Future large-scale, multicenter, prospective studies involving osteoporotic cohorts of diverse ethnicities, including Caucasian and African populations, are urgently needed to validate the universality of these immune inflammation indexes and to explore potential race-specific thresholds. Such studies would provide more reliable evidence for accurate global risk prediction. Furthermore, most of the included research employed retrospective designs, which ma.y not adequately control for selection bias and confounding variables, thereby potentially compromising the robustness of the conclusions. To overcome this methodological limitation, future investigations should prioritize prospective cohort studies. Such studies are pivotal for establishing temporal relationships between immune inflammation indexes and subsequent fracture events, thereby providing a firmer basis for causal inference. Once the predictive value of these indicators is confirmed, research should advance toward interventional trials to translate predictive insights into actionable strategies, ultimately supporting precision prevention and treatment of osteoporosis.

Most studies did not define specific cut-off values for the respective immune inflammation indexes, and among those that did, substantial variability was observed. This inconsistency hampers cross-study comparability and data synthesis, likely contributing to the considerable heterogeneity identified in our meta-analysis. The absence and variability of established thresholds pose significant challenges to transforming these indexes from investigational markers into clinically practical prognostic tools. Without large-scale, validated cut-off values demonstrating definitive diagnostic utility, clinicians cannot reliably identify high-risk patients or make informed clinical decisions based on these markers, as they would when interpreting T-scores in osteoporosis diagnosis. Therefore, concerted efforts are needed to establish standardized threshold systems with strong discriminatory capacity to ensure research consistency and clinical applicability. These thresholds should be stratified by sex, age, and osteoporosis subtype to enhance the precision and relevance of risk assessment and clinical decision-making.

Additionally, the present study suggests that immune inflammation indexes may complement the traditional FRAX model in risk prediction. Whereas FRAX relies primarily on conventional clinical risk factors, inflammatory markers dynamically reflect underlying biological processes directly related to bone resorption. Integrating immune inflammation indexes into existing assessment frameworks may aid in identifying “hidden” high-risk individuals characterized by persistently elevated inflammation despite moderate FRAX scores, thus enabling more refined risk stratification. Future studies should focus on elucidating the biological mechanisms linking immune inflammation indexes with osteoporosis, developing innovative composite predictive models incorporating inflammatory biomarkers, and exploring early intervention strategies for patients with elevated inflammation levels.

## 5 Conclusion

The immune inflammation indexes, NLR, LMR, and SII, exhibit considerable potential in predicting fracture risk among individuals with osteoporosis. Meanwhile, given the limited number of studies evaluating the SIRI, only two in total, its predictive potential requires further validation through additional high-quality research. Subgroup analysis indicates that the prediction performance of these indexes may vary according to the specific type of osteoporosis. Given the methodological limitations, including the predominance of retrospective designs, small sample sizes, geographic homogeneity, and possible instability and publication bias, additional international, multicenter, and prospective studies are urgently required. Such efforts will be essential to validate these indexes as reliable tools for fracture risk prediction, with the ultimate goal of facilitating early identification and timely intervention for high-risk individuals.

## Data Availability

The original contributions presented in the study are included in the article/**Supplementary Material**. Further inquiries can be directed to the corresponding author.
