# Supplementary material for: The predictive value of immune inflammation indexes for the risk of fracture in patients with osteoporosis: a systematic review and meta-analysis
Source: Front Endocrinol (Lausanne). 2025 Nov 3;16:1650895. doi: 10.3389/fendo.2025.1650895 (PMC12620189; doi:10.3389/fendo.2025.1650895)
Supplement: Supplementary file 1 [file DataSheet1.docx]

**Supplementary Table S1** Literature search strategy

Pubmed

(((("Lymphocytes"[Mesh]) OR (((Lymphocyte) OR (Lymphoid Cells)) OR (Lymphoid Cell))) AND (Ratio)) AND (("Fractures, Bone"[Mesh]) OR (((((((((Fracture) OR (Bone Fracture)) OR (Bone Fractures)) OR (Broken Bones)) OR (Broken Bone)) OR (Spiral Fractures)) OR (Spiral Fracture)) OR (Torsion Fractures)) OR (Torsion Fracture)))) AND (("Osteoporosis"[Mesh]) OR ((((((((((Osteoporoses) OR (Age-Related Osteoporosis)) OR (Age-Related Osteoporoses)) OR (Age Related Osteoporosis)) OR (Age-Related Bone Loss)) OR (Age-Related Bone Losses)) OR (Senile Osteoporoses)) OR (Senile Osteoporosis)) OR (Post-Traumatic Osteoporoses)) OR (Post-Traumatic Osteoporosis)))

Embase

((Lymphocytes or (Lymphocyte or Lymphoid Cells or Lymphoid Cell)) and Ratio and (Fractures, Bone or (Fracture or Bone Fracture or Bone Fractures or Broken Bones or Broken Bone or Spiral Fractures or Spiral Fracture or Torsion Fractures or Torsion Fracture)) and (Osteoporosis or (Osteoporoses or Age-Related Osteoporosis or Age-Related Osteoporoses or Age Related Osteoporosis or Age-Related Bone Loss or Age-Related Bone Losses or Senile Osteoporoses or Senile Osteoporosis or Post-Traumatic Osteoporoses or Post-Traumatic Osteoporosis))).af.

Cochrane

((Lymphocytes or (Lymphocyte or Lymphoid Cells or Lymphoid Cell)) and Ratio and (Fractures, Bone or (Fracture or Bone Fracture or Bone Fractures or Broken Bones or Broken Bone or Spiral Fractures or Spiral Fracture or Torsion Fractures or Torsion Fracture)) and (Osteoporosis or (Osteoporoses or Age-Related Osteoporosis or Age-Related Osteoporoses or Age Related Osteoporosis or Age-Related Bone Loss or Age-Related Bone Losses or Senile Osteoporoses or Senile Osteoporosis or Post-Traumatic Osteoporoses or Post-Traumatic Osteoporosis))).af.

Web of Science

((((Lymphocytes) OR (((Lymphocyte) OR (Lymphoid Cells)) OR (Lymphoid Cell))) AND (Ratio)) AND ((Fractures, Bone) OR (((((((((Fracture) OR (Bone Fracture)) OR (Bone Fractures)) OR (Broken Bones)) OR (Broken Bone)) OR (Spiral Fractures)) OR (Spiral Fracture)) OR (Torsion Fractures)) OR (Torsion Fracture)))) AND ((Osteoporosis) OR ((((((((((Osteoporoses) OR (Age-Related Osteoporosis)) OR (Age-Related Osteoporoses)) OR (Age Related Osteoporosis)) OR (Age-Related Bone Loss)) OR (Age-Related Bone Losses)) OR (Senile Osteoporoses)) OR (Senile Osteoporosis)) OR (Post-Traumatic Osteoporoses)) OR (Post-Traumatic Osteoporosis))) (Topic)

Wanfang


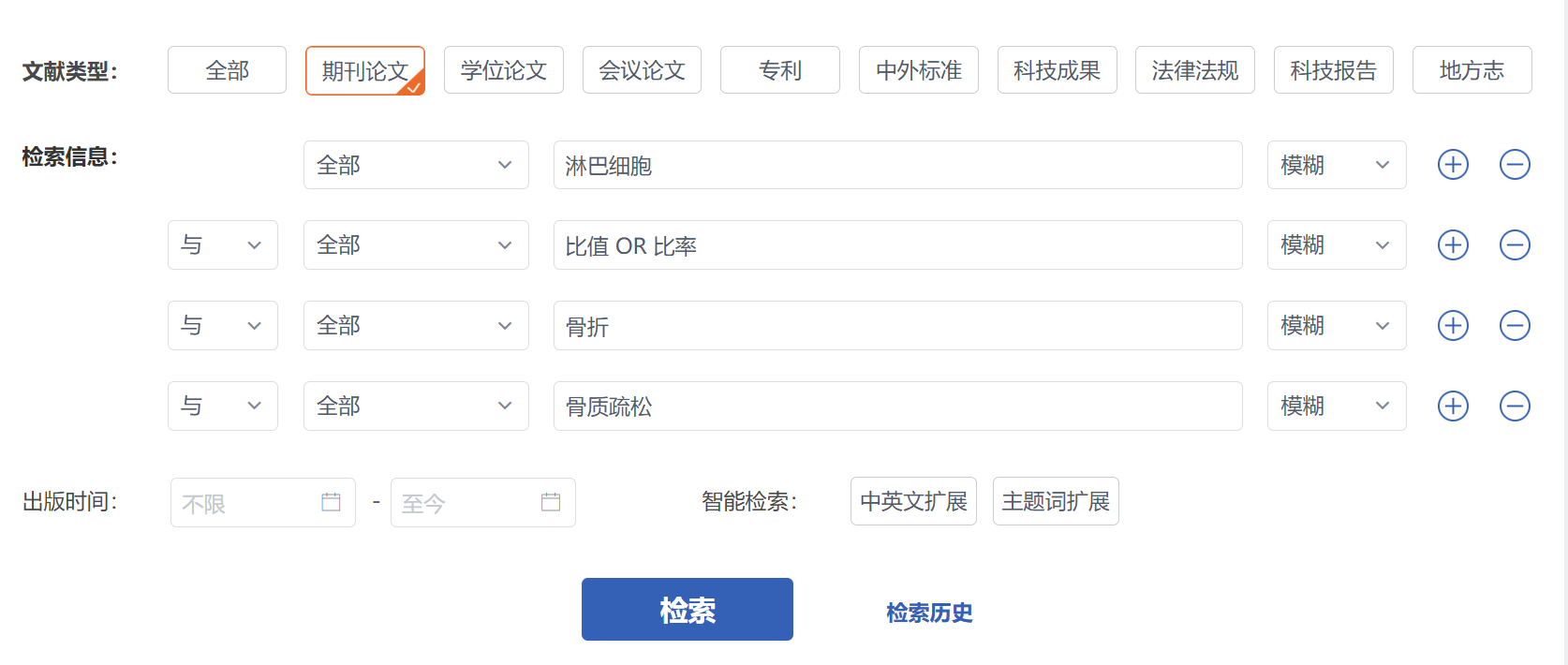


CNKI


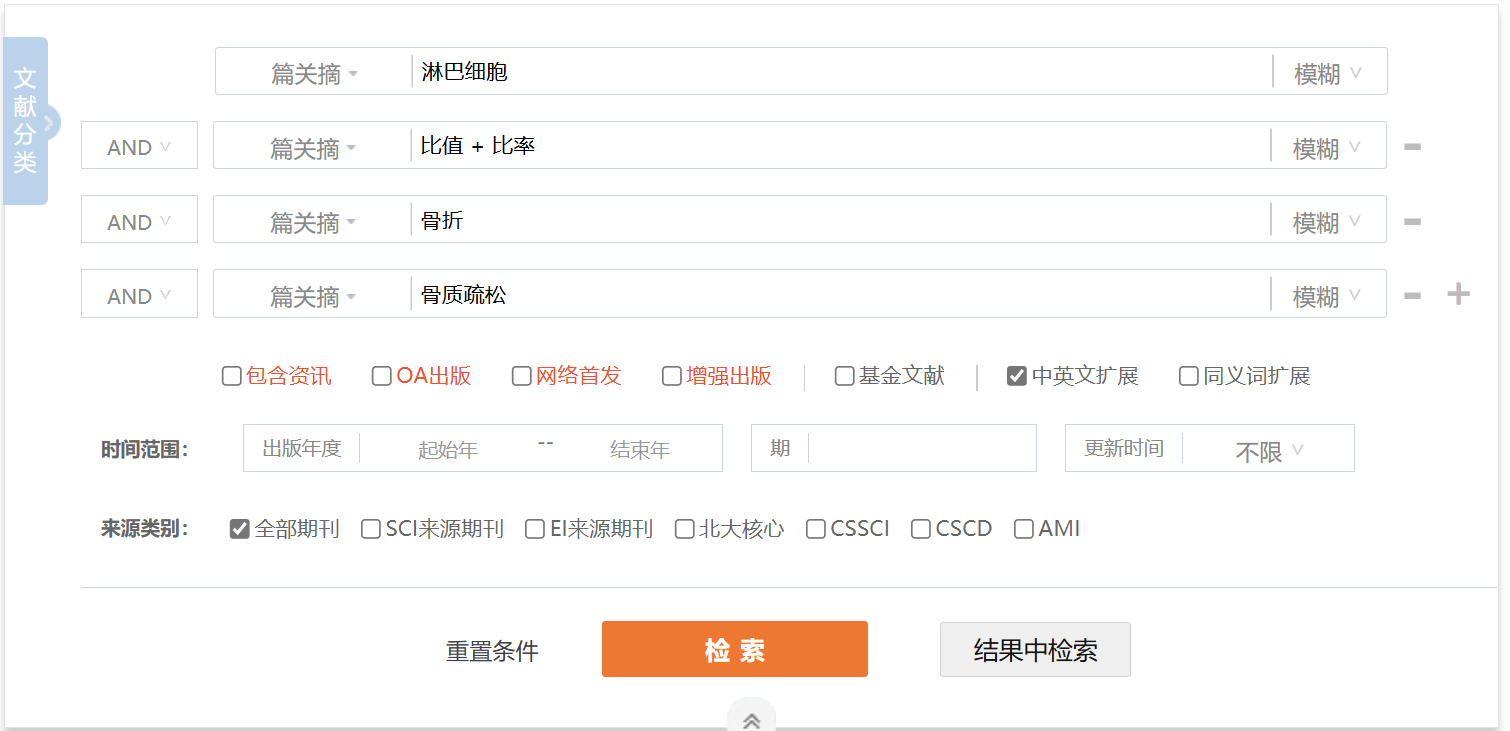


| **Supplementary Table S2.** Quality evaluation of the eligible studies with Newcastle–Ottawa scale.  Study quality of cohort studies | | | | | | | | | |
| --- | --- | --- | --- | --- | --- | --- | --- | --- | --- |
| Study | Selection | | | | Comparability | | Outcome | | |
|  | Representative-ness | Selection of  non-exposed | Ascertainment  of exposure | Outcome not present at start | Comparability on most important factors | Comparability on other risk factors | Assessment of outcome | Long enough follow-up (median≥1 year) | Adequacy  (completeness) of follow-up |
| Li 2023 | - | * | * | * | * | - | * | * | * |
| Song 2022 | - | * | * | * | - | - | * | * | * |
| Fang 2020 | - | * | * | * | * | - | * | * | * |
| Liu 2024 | - | * | * | * | * | - | * | * | * |
| *indicates criterion met; - indicates significant of criterion not met. | | | | | | | | | |
| Study quality of case-control studies | | | | | | | | | |
| Study | Selection | | | | Comparability | | Outcome | | |
|  | Appropriateness of the case determination | Representativeness of the cases | Selection of the control | Determination of the control | Comparability on most important factors | Comparability on other risk factors | Determination of exposure factors | Same method of determination for cases  and controls | Non-response rate |
| Song Y 2022 | * | * | * | * | - | - | * | * | * |
| Chen 2024 | * | - | * | * | - | - | * | * | * |
| Zhang 2024 | * | - | * | * | * | - | * | * | * |
| Fu 2024 | * | * | * | * | - | - | * | * | * |
| *indicates criterion met; - indicates significant of criterion not met. | | | | | | | | | |
